# Dynamics of ferro fluid droplet impact on hydrophobic mesh surface

**DOI:** 10.1039/d5ra02833g

**Published:** 2025-07-30

**Authors:** Bekir Sami Yilbas, Ghassan Hassan, Abdullah Al-Sharafi, Abba Abdulhamid Abubakar, Hussain Al-Qahtani

**Affiliations:** a Mechanical Engineering Department, King Fahd University of Petroleum and Minerals (KFUPM) Dhahran 31261 Saudi Arabia bsyilbas@kfupm.edu.sa +966 3 860 4481; b Interdisciplinary Research Center for Sustainable Energy Systems (IRC-SES), King Fahd University of Petroleum and Minerals (KFUPM) Dhahran 31261 Saudi Arabia; c K.A.CARE Energy Research & Innovation Center Dhahran 31261 Saudi Arabia; d Turkish Japanese University of Science and Technology Istanbul Turkey; e Interdisciplinary Research Center for Advanced Materials, King Fahd University of Petroleum and Minerals Dhahran 31261 Saudi Arabia

## Abstract

Droplet impact has been of interest in various fields extending from environmental science to engineering fields and understanding droplet behavior becomes critical in exploring processes like erosion, painting, oil–water separation, and heat transfer. Droplet impact behaviour can be altered by external influences such as ultrasonic excitations, magnetic fields, thermal radiation, and similar. In the present study, impact dynamics of water-ferro particles mixed droplet over hydrophobic mesh surfaces under magnetic influence are investigated and dynamic characteristics are assessed for various ferro-particles concentrations and mesh sizes. Droplet restitution, rebound, implement, and newborn droplet properties are analysed and explored experimentally. It is demonstrated that Weber number, mesh size and ferro particle concentration in droplet fluid have significant effect on the droplet behaviour over hydrophobic mesh. Increasing Weber number (We > 12) lowers the restitution coefficient, which becomes notable for large mesh aspect ratios. Droplet contact duration on mesh surface reduces with increasing Weber number while it reduces with ferro-particle concentrations. Volume of droplet fluid penetration enhances with ferro particle concentration and Weber number. Similarly, number of fragments of penetrated fluid increases with particle concentration and Weber number demonstrating the strong magnetic influence on the mechanism of droplet fluid fragmentation.

## Abstract 2

Experimental processes involved with droplet impact analysis.

## Graphical abstract

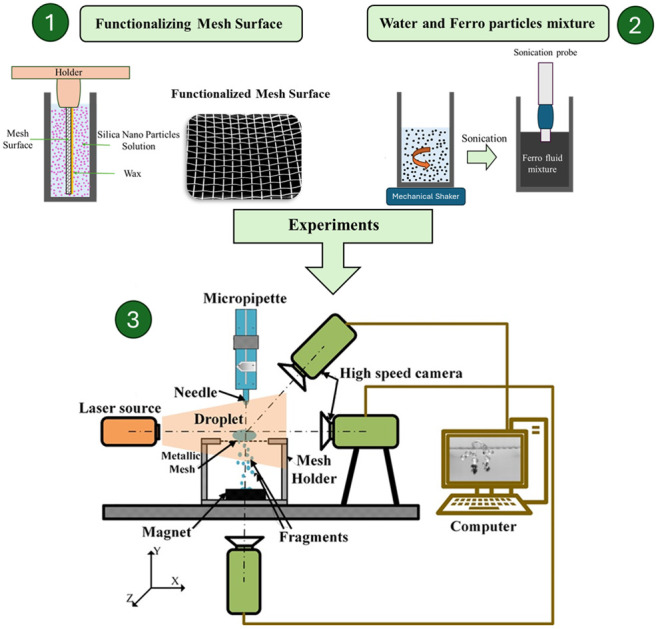

## 1 Introduction

Application of liquid droplet impact on surfaces spans over several areas of engineering varying from biomedical^1^ to surface self-cleaning.^2^ Surface hydrophobicity becomes critically important for impacting droplet behaviour since hydrophobic surface enables to alter droplet deformation, retracting and rebounding behaviour after the impact. This occurs because of the combination of low dissipative energy under low adhesion forces and high retention droplet energy after impact.^3^ The impact dynamics are also influenced by external effects such as magnetic, ambient conditions (temperature, pressure), flow, surface structure, and similar. Impact surface magnetic influence remains important if the droplet fluid has the ferro-fluid characteristics while surface geometry becomes important if the impacting surface is meshed. The impact of ferro-fluid droplet could be altered by introducing magnetic effect and newborn droplet could be created depending on the position and strength of the magnet.^4^ For droplet impacting on meshed hydrophobic surfaces, droplet fluid penetration and separation from the surface can be altered *via* changing the droplet volume.^5^ However, examining the combined effects of magnetic influence and the surface structure, such as mesh, on impact dynamics of droplet can be fruitful for various applications. Consequently, investigation of impacting droplet dynamics including both effects becomes essential.

Interfacial resistance between the liquid and hydrophobic surfaces remains smaller than that of the hydrophilic surfaces. This in turn minimizes the liquid spreading over the surface. Although surface hydrophobicity has been studied extensively, hydrophobic meshes and liquid droplet interactions in terms of droplet mobility and impact characteristics have become a recent interest of many researchers. The study on the droplet mobility on hydrophobic mesh, in relation to self-cleaning applications, demonstrated that hydrophobic meshes are one of the methods to increase the droplet mobility in terms of speed and acceleration on surfaces.^5^ However, the mesh size, as compared to droplet size, remains critical to maximize the droplet mobility. The droplet inflection and bulging alter the force balance on the surface and gravitational force created in the inflected part of the droplet has an adverse effect of the droplet mobility. From this end, as the water droplet impacts a hydrophobic mesh, its behavior is influenced not only by surface hydrophobicity but also by the size and structural characteristics of the mesh. Unlike solid hydrophobic surfaces, where droplets only undergo stretch, contract, and rebound, a hydrophobic mesh can either repel the droplet or allow it to pass through, depending on mesh size, impact velocity, and surface tension. As the hydrophobic mesh size is smaller than the droplet diameter, the droplet may spread momentarily before recoiling and bouncing off.^6^ However, if the pores are large enough, the droplet may penetrate through the mesh, breaking into smaller droplets due to capillary and gravitational forces.^5^ In addition, depending on the hydrophobic mesh flexibility rebounds and droplet behavior on the mesh surface changes significantly. As the mesh oscillates with a fixed frequency, the droplet retraction time was influenced by mesh frequency and amplitude and penetration depth of the droplet.^7^ The mesh conditions, such as prewetted surface by residues of the early impact, can also influence the droplet behavior on the impacted surface. It was demonstrated that as the droplet Weber number increases the transition from no penetration to complete penetration into mesh.^8^ In the applications of oil–water separation, dynamic behavior of droplet on oleophilic meshes becomes critically important for water separation process in terms of controlling the liquid velocity, imbibition, and liquid pinching over the surface. It was demonstrated that during the partial imbibition process, the maximum spreading ratio enhances with Weber number; however, opposite occurred during the separation phenomena and the influence of Weber number becomes less on the separation.^9^ Liquid imbibition/penetration, mainly, depends on mesh pore size, wettability, and properties of droplet liquid impacting over the mesh surface. For quasi-penetration process, it was shown that pore size do not influence the penetration pressure but do influence the pressure at which the liquid penetration ends. This is because the droplet liquid spreads out and increases the wetted area while causing capillary pressure resisting penetration.^10^ Modifying the fluid properties by adding soluble materials alters the dynamics of impacting droplet on meshes. From this end, it was demonstrated that droplet formed with the mixture of water and polyethylene oxide (PEO) alters droplet impact properties on hydrophobic mesh surfaces. In this case, droplet rebound occurred in a small velocity range and the low size filaments can connect to the droplet surface while creating some small adhesion between the polymeric fluid and the hydrophobic mesh.^11^

Superhydrophobic meshes, often inspired by biological structures like spider silk or plant leaves, enhance water repellency by utilizing micro- and nanoscale textures that trap air within the mesh pores. This enables the droplet either to undergo complete bouncing or rolling away over the mesh surface with low adhesion. This process becomes particular interest for various applications including fog harvesting, self-cleaning, and biomedicine. As the ferrofluid droplets impact a super hydrophobic mesh under the influence of a magnetic field, their impact dynamics of droplet becomes substantially complex because of the influence of dynamic forces created among surface tension, hydrophobicity, mesh porosity, and magnetism. Since ferrofluids contain suspended magnetic particles in the liquid, the particles are attracted by the magnetic field. The impacting droplet can possibly bounce off, break off on the hydrophobic surface depending on the strength and orientation of the magnetic force created.^4^ In some cases, the droplet can notably deform prior to rebounding and a magnetic force may pull the droplet towards the hydrophobic surface while altering the impact dynamics.^4^ In addition, the collective influence of these forces results in an equilibrium droplet shape, which depends on the magnitude and direction of the forces; hence, the ferrofluid droplet shape resembles the different equilibrium states.^12^ Ferrofluid droplet impact on hydrophobic surfaces creates a magneto-elastic effect while causing droplet rebound heigh suppression. However, addition of polymers to the droplet fluid can further alter the droplet dynamics including the rebound height. It was demonstrated that ferrofluid droplet with inclusion of polymers gives rise to rebound suppression at low Bond and Weber numbers, however, keeping Weber number constant while increasing ferro particle concentration, rebounding initiates early due to the influence of the magnetic field.^13^ The spreading dynamics of a droplet on impacted hydrophobic surface becomes different as the magnetic flux is kept constant. In this case, a steady-state droplet shape with a reduced base diameter is observed and, at the end of the spreading, apex height of the droplet increases at higher magnetic Bond numbers.^14^

The ferrofluid impact on hydrophobic meshes under magnetic influence has considerable implications in practice, since the process enables the development of tunable filtration membranes,^15^ adaptive liquid-repellent surfaces,^16^ and precise fluid handling systems.^17^ Although numerous studies have been carried out to explore the ferrofluid droplet impact on hydrophobic surfaces, the impact dynamics needs further examinations to explore interactions of ferroparticles and magnetic field and their influence on spreading, retraction, and rebounding characteristics of the droplet on hydrophobic meshes. In the present study, ferrofluid droplet impacting on the hydrophobic meshes and impact characteristics of droplet are examined under the constant magnetic flux. The droplet inflection and separation from the mesh surface are formulated and meniscus height is predicted from the force balance. The influence of Weber and magnetic Bond numbers on the impacting droplet behavior is analyzed for different ferroparticle concentrations.

## 2 Experimental

### 2.1 Ferro particles and meshes

Ferro particles (Fe_3_O_4_, Sigma Aldrich)) about 50 nm were mixed with water at concentrations of 0.05% (wt%) and 0.005 (wt%). To achieve a uniform particle distribution in the mixture, ultrasonic shake was applied over nine hours. Since suspended ferro particles were prone to destabilization and form sediments under various forces such as van der Waals, gravitation, buoyancy, and magnetic, the stability of any colloidal suspension was assured through zeta potential assessment. The zeta potential was estimated as −30 mV, which is consistent with the previous study.^18^ Tests were conducted towards further assessing ferro particles suspension. The findings reveal that particle suspension in water was observed to be stable over 500 hours. The mixture density, viscosity, and surface tension were measured, which are 1080 kg m^−3^, 1.35 mPa s, and 0.067 N m^−1^, respectively. A steel mesh with wire diameter of 400 μm is hydrophobized through depositing the functionalized particles *via* deep coating technique. The mesh is square in geometry with a fixed screen aperture ratio for each mesh sample used in the experiments. The Aperture Ratio 
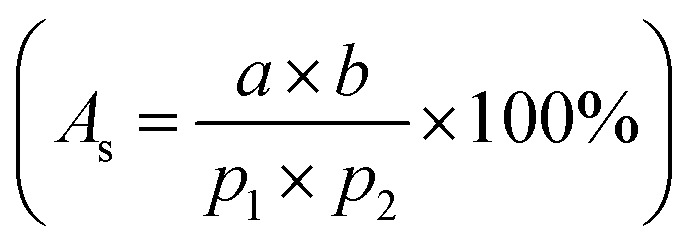
 is defined through the screen area (*a* × *b*, here *a* and *b* are the square mesh lengths and *a* = *b*) over the pitch area (*p*_1_ × *p*_2_, here *p*_1_ and *p*_2_ are the mesh size including the mesh wire diameter and *p*_1_ = *p*_2_).

### 2.2 Mesh wetting state

**Fig. 1:**
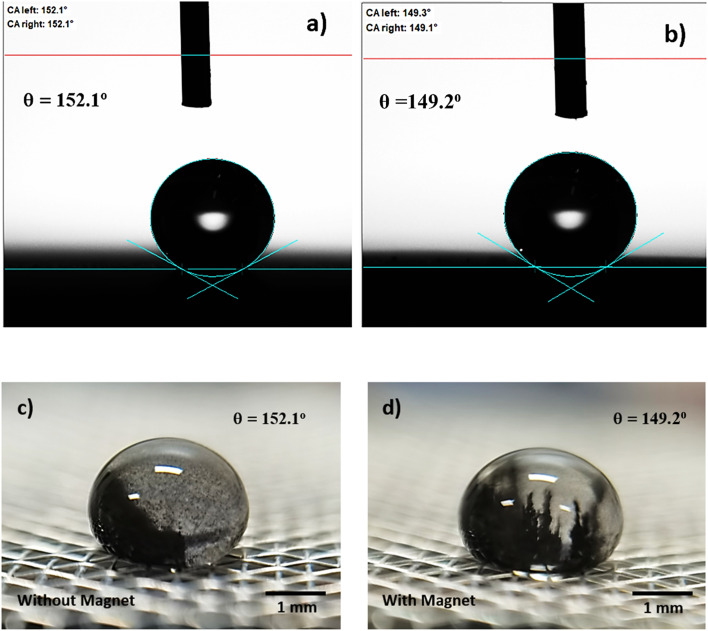
Goniometer and optical images of droplet: (a) goniometer image without magnet, (b) goniometer image with magnet, (c) optical image without magnet (ferro particles are loose in droplet fluid), and (d) optical image with magnet (ferro particles are aligned in the direction of magnetic field make columnar like structures).

The mesh was hydrophobized by functionalized silica particles deposition similarly as described in the previous studies.^19,20^ The wetting characteristics of mesh surface differs from the plain surfaces and contact angle is influenced by the mesh screen size. The previously formulated contact angle formulation is adopted for the contact angle calculations, if mesh is considered as the isotropic structure,^21^ the apparent droplet contact angle (*θ*_app_) becomes:1
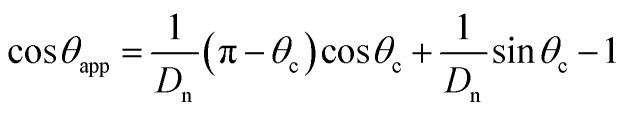
Here, *θ*_c_ is droplet contact angle at flat surface, 
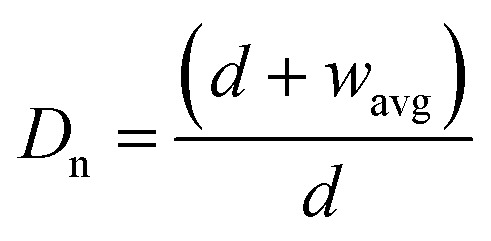
 is normalized mesh average pitch, *d* is wire diameter, *w*_avg_ is aperture average width (
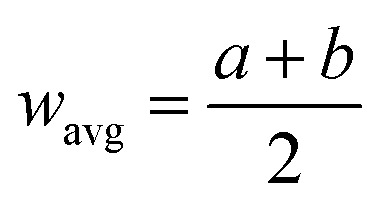
, *a* and *b* are mesh cell wire spacing, here *a* = *b*). In addition, the magnetic force can alter the droplet contact angle. A study is extended to examine the influence magnetic force on droplet contact angle because of change of the mixture surface tension. The equivalent surface tension due to magnetic effect becomes:^4,22^2
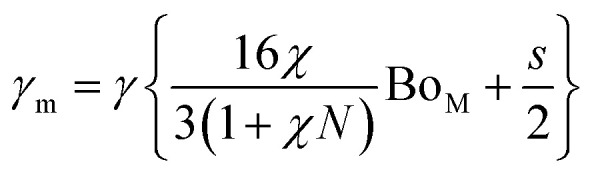
Here, *χ* is susceptibility, *N* is demagnetizing, Bo_M_ is Bond number, *s* is surface area scale (
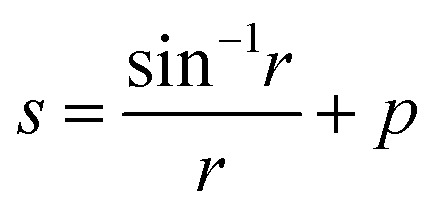
 here 
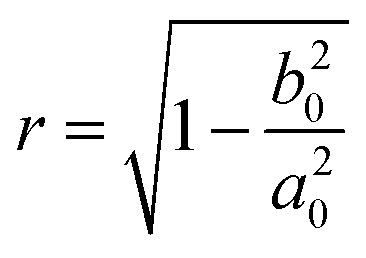
 is droplet surface eccentricity and 
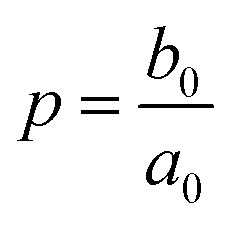
 is length scale ratio, *a*_*0*_ is major length, and *b*_0_ is droplet minor length). After observing elliptic like droplet geometric feature under the magnetic effect *χ* becomes *χ* = 1.2 (ref. 23) and *N* = 0.5 (ref. 24) and measuring 
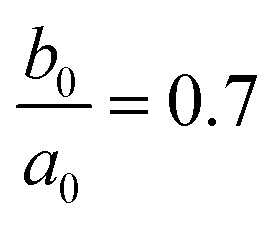
, Bo_M_ ∼ 0.1 (because of low Ferro particle concentration), surface tension ratio reference to water 
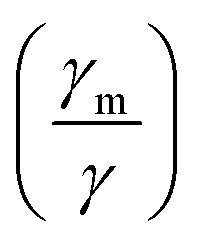
 becomes 1.025, *i.e.* the magnetic force slightly alters the surface tension, which causes slight alteration of droplet contact angle. The mesh wetting status was assessed using Goniometer and droplet contact angle was measured at several locations on the mesh surface and it was observed that droplet contact angle only changed about 1% over the hydrophobized entire mesh surface. The droplet contact angle was ∼152° ± 1° and the hysteresis is 3.5° ± 2°. Fig. 1a and b shows droplet contact angles while Fig. 1c and d show droplet images on the mesh surface without and with magnetic effect, respectively. A strong magnet (NdFeB, K&J Magnetics Inc., USA) having magnetic strength of 11.806 × 10^5^ was incorporated and located below the impacted hydrophobic mesh.

### 2.3 High speed recording

**Fig. 2:**
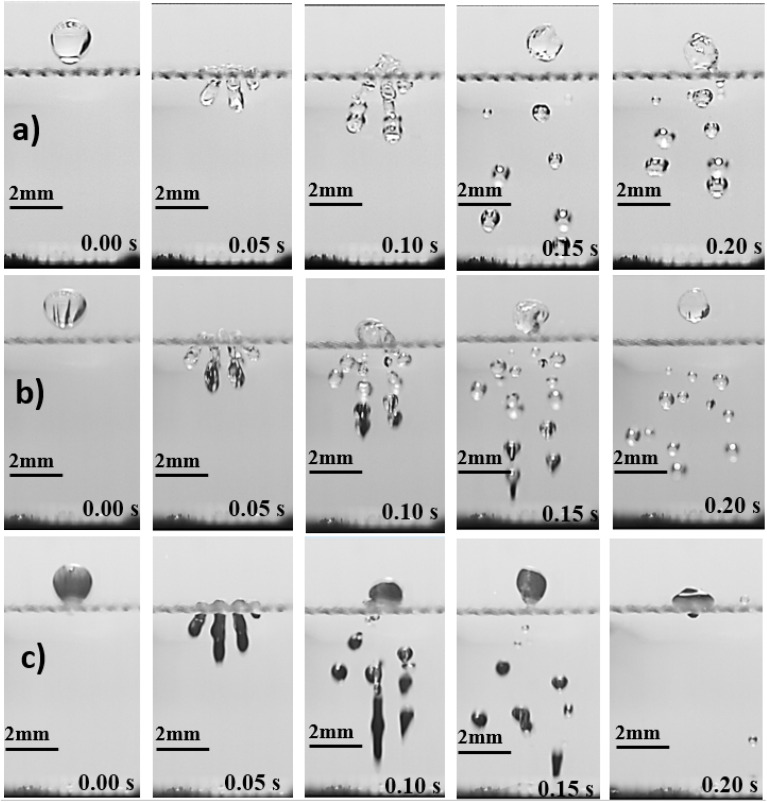
Highspeed camera images impacting droplet: (a) pure water, (b) ferro particles mixed droplet fluid (concentration = 0.005% wt), (c) ferro particles mixed droplet fluid (concentration = 0.05% wt). Mesh ratio (*A*_s_) is 61.2%.

Fig. 2 demonstrates the schematic of experiment. A camera operating at high speed (5000 fps) and the image resolution was 1280 × 800 pixels (each of 14 μm × 14 μm). To analyze the images a tracker program was used, and uncertainty analysis was conducted, like the previous study,^3^ to assure the uncertainty involved in data analysis, which was determined about 3.2%, which was similar to that reported in the early work.^25^

## 3 Results and discussion

### 3.1 Ferroparticles and magnetic influence

Ferro-liquid droplet impacting on a hydrophobized mesh surface is investigated after considering the magnetic effect. The force, due to magnetic field, acting over the droplet because of ferro-particles can be formulated in line with the previous study:^26^3
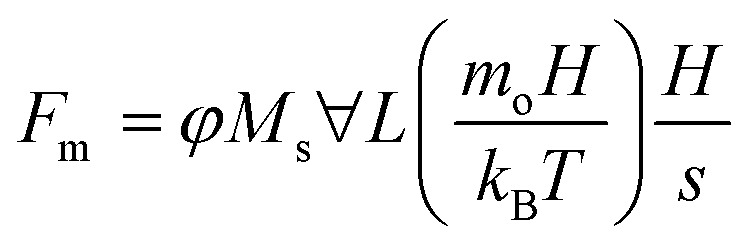
Here *φ* is particle volumetric concentration, which is 
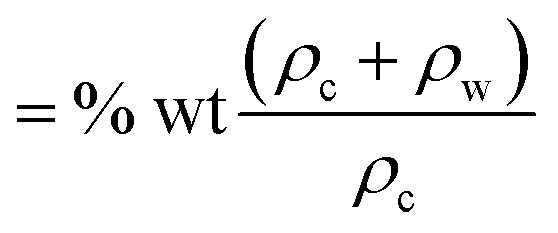
, *ρ*_c_ is particle density, and *ρ*_w_ is density of water, *M*_s_ is saturated magnetization per unit volume, *H* is strength of magnetic field, *m*_o_ is moment due to particles under magnetic influence, *T* is temperature, *k*_B_ is Boltzmann constant, *s* is spacing among particle and magnet, and 
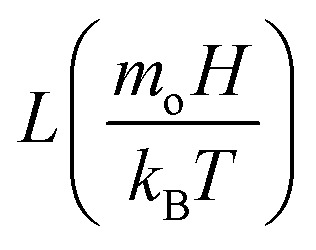
 is the Langevin function 
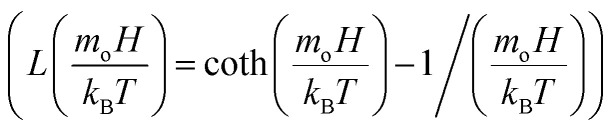
. Given that the argument of the Langevin function is approximately 
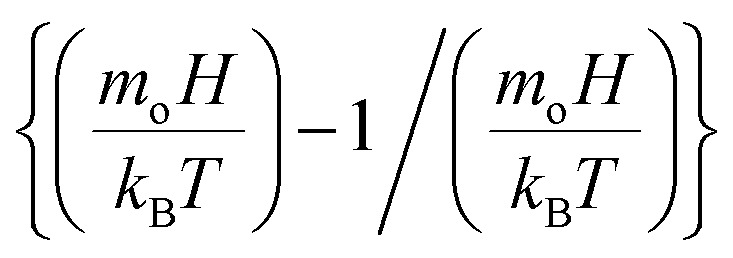
 is about ∼104, its value can be assumed to be unity under the experimental conditions, *i.e.*
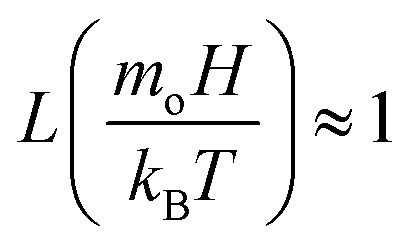
. Introducing *ρ*_c_ = 5150 kg m^−3^,^27^*ρ*_w_ = 980 kg m^−3^, *M*_s_ = 6.65 × 10^−3^ mT (for ferro-particles), and 0.055 (wt%) concentration, *H* = 6.95 × 10^4^ A m^−1^ magnetic field strength, magnet surface and droplet spacing of *s* ≈ 20 mm, the force due to magnetic effect becomes ∼2.3 × 10^−5^ N. As droplet nears the impacting area, the force due to magnetic field increases because of the reduced separation distance (*s*), as described in eqn (3). Arranging impact distance as droplet and impact surface spacing (*s*), droplet diameter of ∼4.5 mm just before impact, magnetic field strength can be determined as *H* ≈ 6.98 × 104 A m^−1^ and the force becomes ∼5.9 × 10^−5^ N. Moreover, capillary force (*F*_*γ*_) acting on particles towards sustaining particles in droplet fluid becomes,^4,26^*i.e.*:4
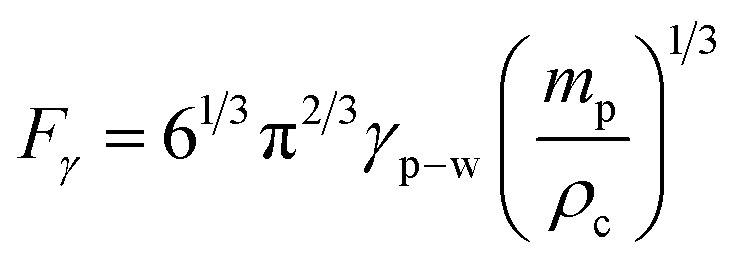
Here, *m*_p_ is particle mass. Ferro-particles get clustered in droplet fluid because of magnetic field, hence, particles with 0.055% (% wt) concentration, clustered particles mass inside 20 μL droplet yields ∼1.52 × 10^−6^ kg. Interfacial tension *γ*_p−w_ is 0.126 N m^−1^, The interfacial force becomes *F*_*γ*_ = 1.47 × 10^−4^ N. Force acting over particles can be determined from eqn (4), which is ∼2.3 × 10^−5^ N. Hence, force due to interfacial tension becomes larger than magnetic force which gives rise to retaining of particles inside droplet fluid, *i.e.* particles retain in the fluid at impact rather than pulled off from droplet fluid under magnetic influence. Therefore, forces of gravity (∼1.47 × 10^−5^ N) and magnetic (∼2.3 × 10^−5^ N) acting on particles cause particle settlement at droplet bottom after the impact.

### 3.2 Penetration of impacting ferrofluid droplet into meshes

As droplet impacts into hydrophobized metallic meshes, impalement of the droplet fluid into the gaps occurs due to force created due to dynamic pressure (*p*_d_ = ½*ρv*_i_^2^), water hammer pressure (*p*_wh_ = 1.41*ρcv*_i_) and the capillary pressure 
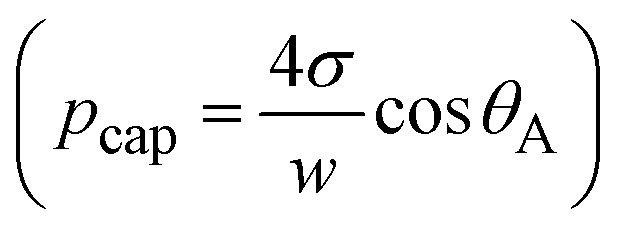
.^28–30^ In the case when *p*_d_ + *p*_wh_ ≥ *p*_cap_, liquid impalement is resulted and a meniscus forms at the droplet-mesh screen interface However, as the sum pf dynamic and water hammer pressures becomes much larger than the capillary pressure (*p*_d_ + *p*_wh_ ≫ *p*_cap_), then, the liquid meniscus penetrates deeper into the mesh screen. As the penetration depth becomes comparable or larger than mesh wire diameter, droplet can undergo fragmentation. Therefore, the liquid volume infused is estimated by a volume of half ellipsoid and the droplet meniscus height is formulated from the force balance along impact axis, *i.e.*:

5 *γ*_g_π*a*^2^*h*_d_ + *γ*_g_Δ*V* + *P*_eff_π*a*^2^ = 0

Here: *γ*_g_ is liquid specific weight, *h*_d_ is droplet height corresponding to a maximum spreading, *F*_*γ*_ is force due to surface tension, Δ*V* is liquid inflection volume, 
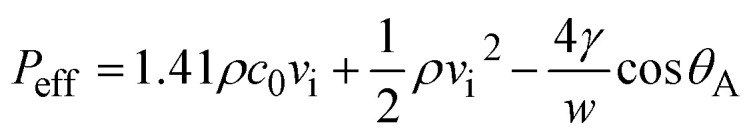
 is effective pressure upon impact, *c*_0_ is the sound speed of sound, *γ* is surface tension, *w* is wire spacing distance and *θ*_A_ is the advancing contact angle, which can be expressed as the cone angle of the volume of penetrated fluid into mesh screens.^31^ Hence, eqn (5) can be expressed as:6
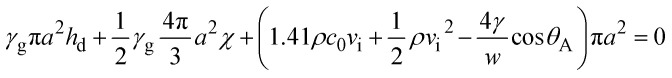


However, 
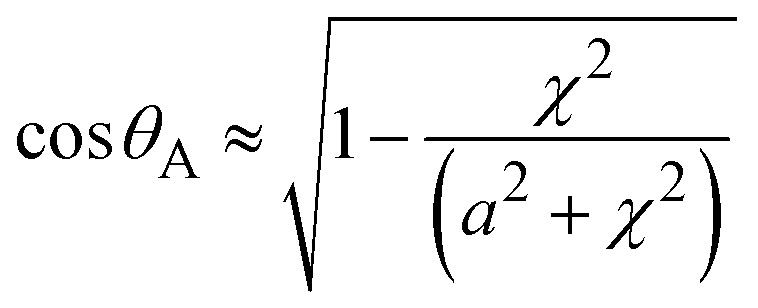
, the meniscus height (*χ*) becomes:7
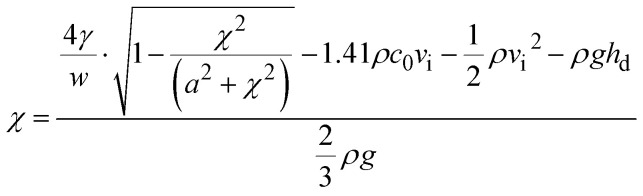


**Fig. 3:**
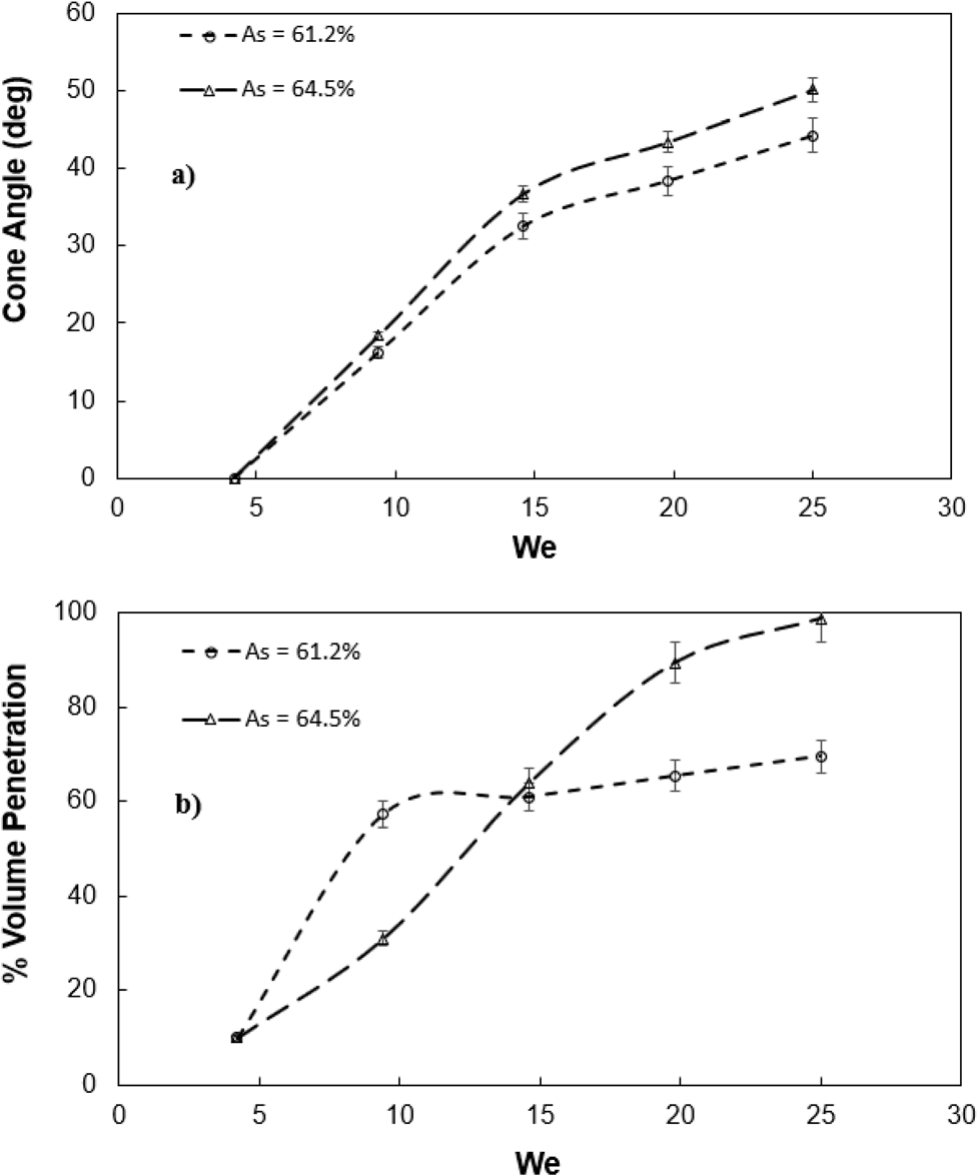
Characteristics of droplet fluid penetrated in hydrophobic mesh: (a) penetrated fluid cone angle with Weber number, and (b) volume percentage of droplet fluid penetrated with Weber number. As represents mesh aperture ratio 
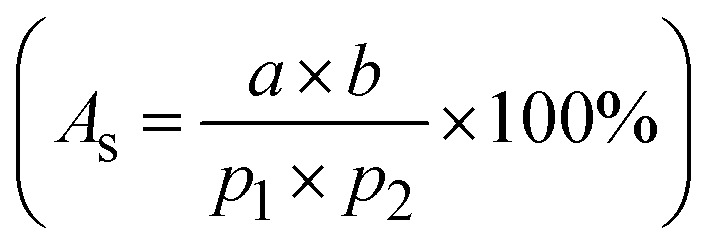
 is defined through mesh screen area (*a* × *b*, here *a* and *b* are the square mesh lengths and *a* = *b*) over the pitch area (*p*_1_ × *p*_2_, here *p*_1_ and *p*_2_ are the mesh size including the mesh wire diameter and *p*_1_ = *p*_2_).

Eqn (7) can be solved iteratively to obtain the meniscus height. Fig. 2 shows high speed camera images of droplets impacting on mesh surfaces with and without ferro particles while Fig. 3a shows the cone angle (*θ*_A_) of the droplet penetrated the mesh screen with Weber number. In addition, the percentage of liquid volume impalement into mesh with Weber number for two mesh screen ratios is shown in Fig. 3b. The droplet liquid impalement is evident for both water and ferro particle mixture droplets. Increasing Weber number enhances both cone angle and percentage of liquid penetration into meshes. Since Weber number represents the ratio of inertial force over the surface tension force and impacting droplet diameter remains same, increase of Weber number resembles enhancement of inertia force rather than reducing surface tension force on impacted surface onset of impact. Hence, increasing inertial force causes large increase of cone angle and significant amount of fluid volume penetrating into the mesh screens. However, the variation of cone angle and percentage of liquid volume penetration with Weber numbers is non-linear. In this case, the sharp increase replaces with a gradual increase at about Weber number 15 and beyond. Although two mesh sizes with close aperture ratios are presented, the behavior of percentage of volume impalement changes considerably, particularly after Weber number of 8 and more. This indicates that the percentage of liquid impalement across mesh screen is critically dependent on mesh size and even slightly small meshes fluid retaining across the mesh screen due to capillary force creates the blockage effect while blocking the volume of impalement fluid flow. It is noted that the mesh area conforms to a square and for large mesh aspect ratios 
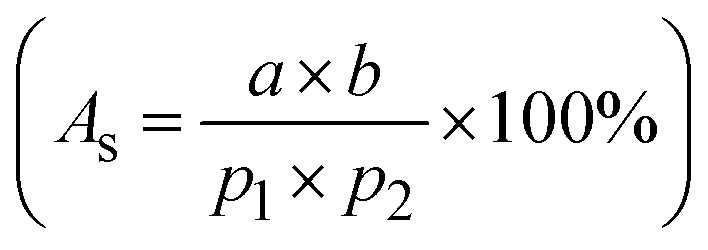
, mesh wire diameter increases, *i.e.* increase in *p*_1_ × *p*_2_ becomes more than that of *A*_s_ = 64.5% than *A*_s_ = 61%, which in turn increases the mesh screen area notably for *A*_s_ = 64.5% mesh. This causes an increase of capillary force created around the mesh wire while influencing the droplet liquid flow into the mesh screen.

### 3.3 Assessment of droplet restitution, spreading and fragmentation

On the other hand, energy loss related to impacting droplets is contended with frictional, formation of fragmented droplets, and deformation work^2^ and energy balance of droplet over the impacted surface can be expressed as:

8 *E*_p1_ + *Ω*_1_ = *Ω*_2_ + *W*_net_ + *E*_p2_

where *E*_p1_ = *ρ*∀_i_*v*_i_^2^ is droplet potential energy just prior to impact, *E*_p2_ = *ρ*∀_f_*v*_r_^2^ is droplet potential energy corresponding to peak rebound height, *v*_i_ is velocity at impact, *v*_r_ is rebound velocity, 
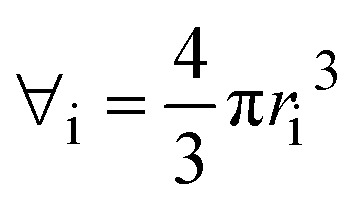
 is volume of droplet before impact, 
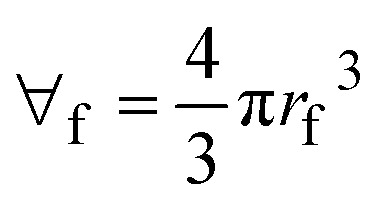
 is droplet rebounding droplet volume, *Ω*_1_ is droplet surface energy before impact, *Ω*_2_ is droplet surface energy at spread over impacted surface, *W*_net_ is energy dissipated during droplet spreading, impalement and fragmentation on meshes, and *ρ* is fluid density. Net energy dissipation can be categorized into:

9 *W*_net_ = *W*_d_ + *W*_v_ + *W*_fr_

Here, *W*_d_ is deformation work during spreading, *W*_v_ viscous dissipation because of interfacial friction and *W*_fr_ is energy dissipation because of droplet fragmentation. Moreover, surface energy can be expressed as:^32^

10 *Ω*_1_ = π*d*_i_^2^*γ*

and11
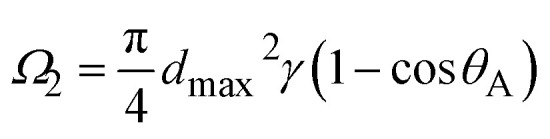
where *d*_max_ and *d*_i_ are maximum and initial droplet diameters respectively, *γ* is surface tension and *θ*_A_ is advancing contact angle.

Work of deformation due to spreading and retraction on impacted surface is:

12 *W*_d_ = 2∀_avg_(*p*_im_ − *p*_rb_)

where, 
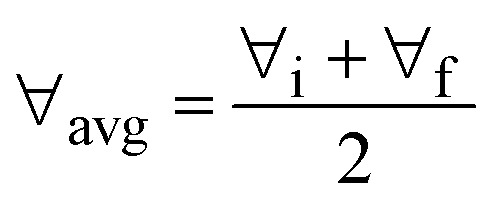
 is droplet average volume during spreading, impalement and fragmentation, 
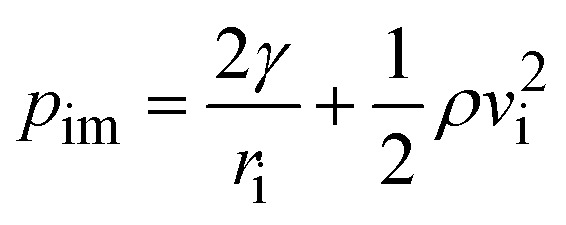
 is droplet impact pressure, 
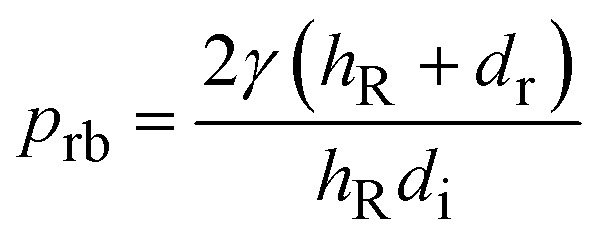
 is retraction pressure, 
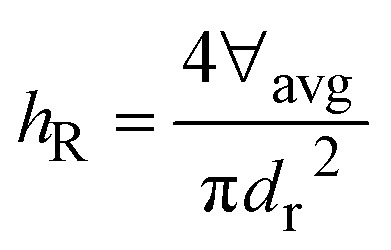
 is retraction height, and *d*_r_ is retraction diameter.

Viscous dissipation is constituted into two categories: (i) *W*_v1_ is related to friction at droplet-mesh interface, and *W*_v2_ is at mesh interior walls, which is considerably small as compared to *W*_v1_ and can be ignored. Viscous dissipation becomes:13
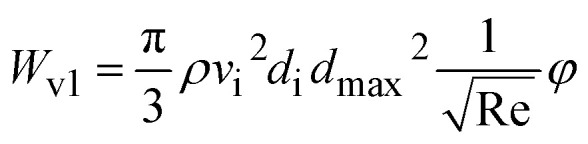
where *φ* is solid fraction and corresponding to ratio of projection area covered by pillars at the surface over surface projected area, and Re is the Reynolds number.^32,33^

Energy dissipated because of formation of droplet fragments is:14
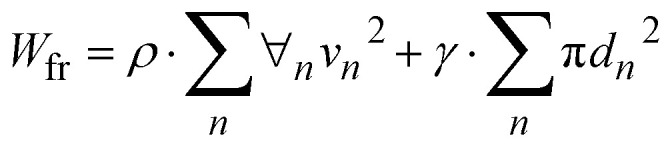
where *ρ* is density, ∀_n_ is individual volume of fragmented droplets, *v*_n_ is the fragmented droplet velocity and *d*_n_ is fragmented droplet diameter. The energy dissipated can influence the physical size of droplet spreading, retraction, and rebounding, which reflects on the magnitude of the spreading rate and the restitution coefficient. The coefficient of restitution can be expressed as in terms of velocity ratio, *i.e.*:15
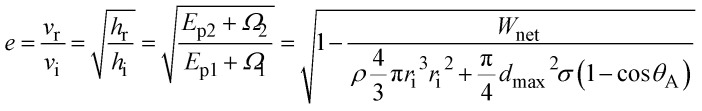


**Fig. 4:**
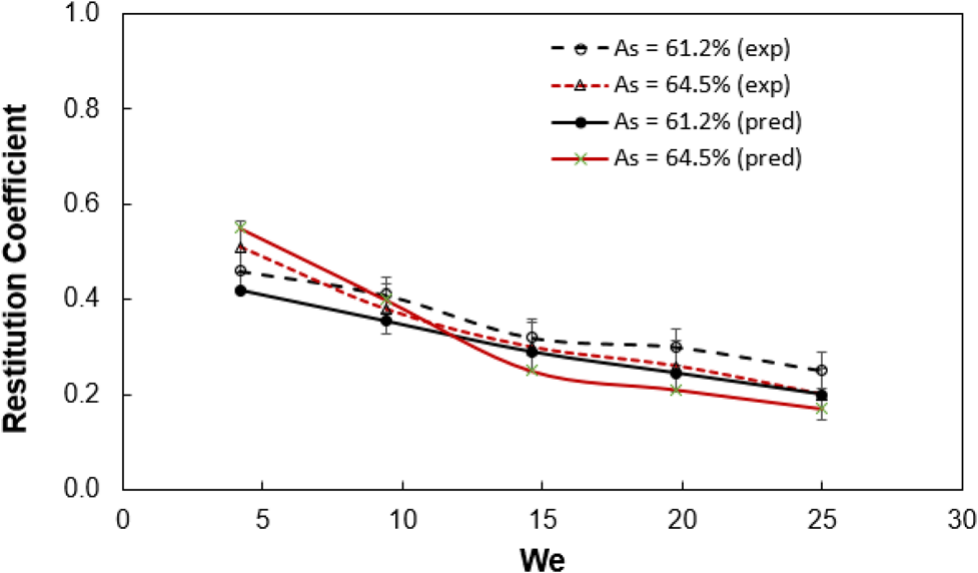
Restitution coefficient of impacted droplet obtained from experiment and predictions for two different mesh aspect rations (*A*_s_).

Fig. 4 shows restitution coefficient predicted from eqn (15) and obtained from experiments for two mesh sizes. It is evident that predictions agree well with the experiments and small variations are related to the assumptions made in the formulation of restitution coefficient due to simplicity. Moreover, increasing Weber number lowers the restitution coefficient for both mesh sizes. This increasing Weber number enhances liquid impalement into meshes and fluid droplet velocity reduces significantly on the surface due to the associated losses, which is unlike the cases for non-meshed hydrophobic surfaces,^4^*i.e.* it is worth mentioning that decrease in restitution coefficient is gradual with Weber number for smooth hydrophobic surfaces.

**Fig. 5:**
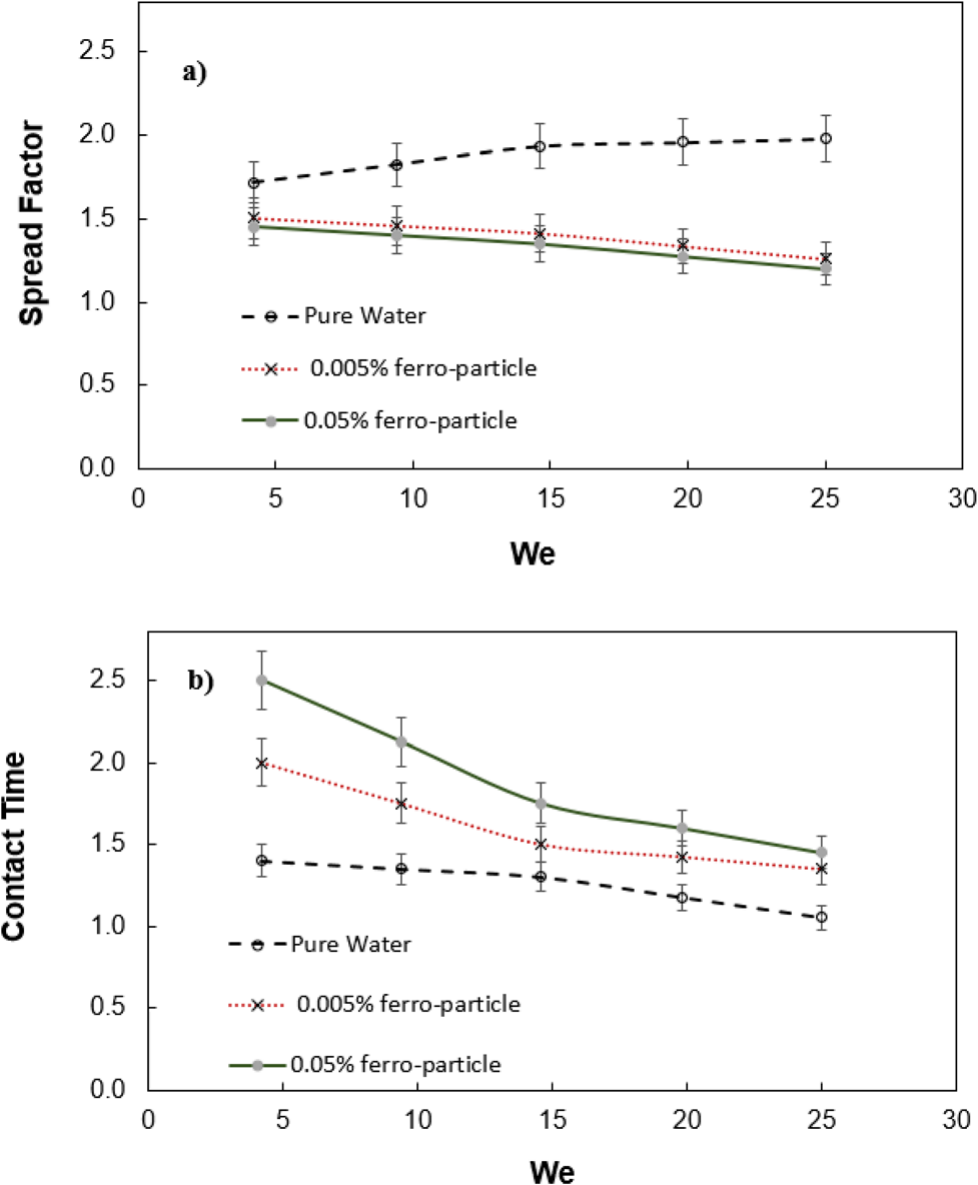
Droplet spread and contact properties at mesh surface with Weber number: (a) spread factor, and (b) contact time (time is normalized by the capillary time, 
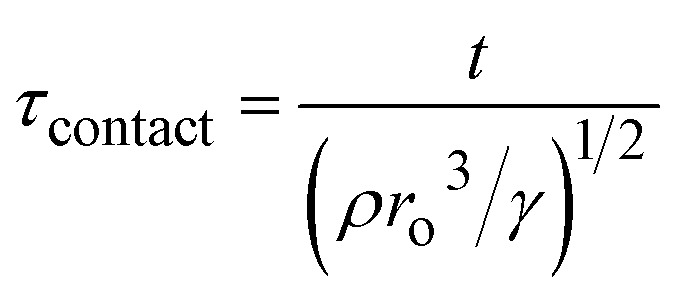
, here *r*_0_ is the droplet radius prior to impact and *γ*is surface tension).

**Fig. 6:**
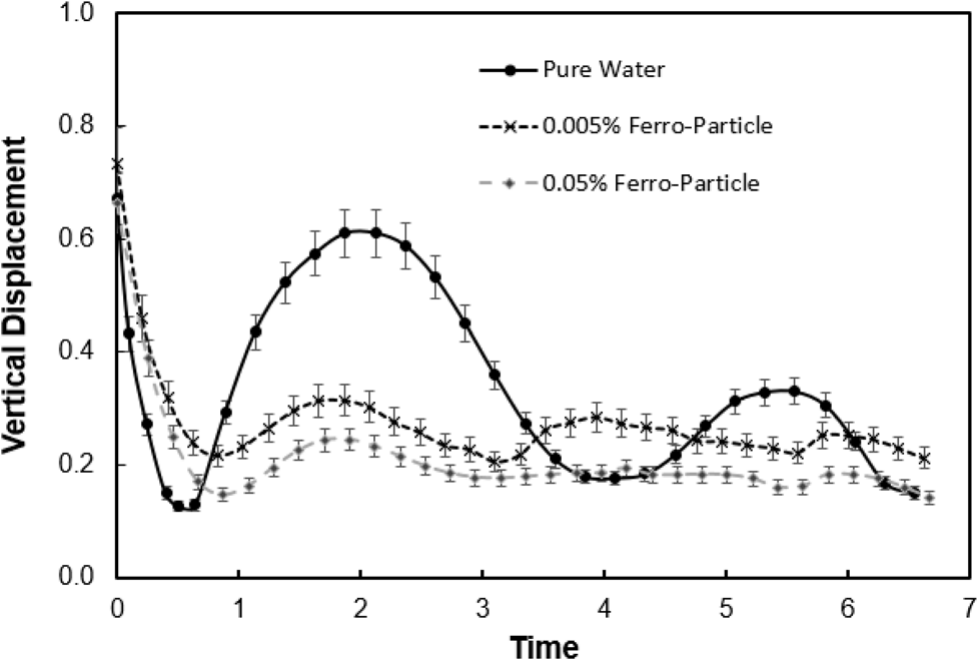
Vertical displacement of rebounded droplet with dimensionless time (time is normalized by the capillary time, 
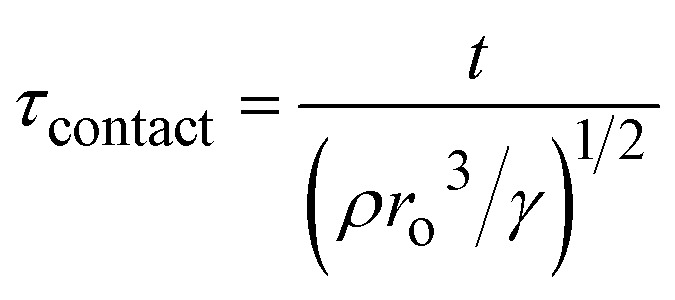
, here *r*_0_ is the droplet radius prior to impact and *γ* is surface tension).

Fig. 5a shows spread factor (
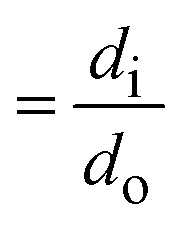
, where *d*_i_ and *d*_o_ before impacting and maximum droplet diameters on mesh surface, respectively) with Weber number for droplet with inclusion of ferro particles while Fig. 5b demonstrates the droplet contact time with Weber number for different concentrations of ferro particle and pure water. The contact time is defined through 
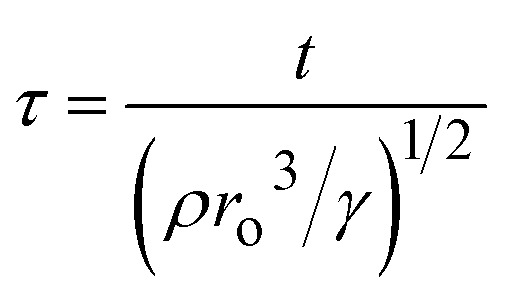
, here *r*_0_ is the droplet radius prior to impact and *γ*is surface tension. Spread factor gradually decreases with Weber number for ferro particles included however, opposite is observed for pure water. This is because of the magnetic field, which influences the ferro particles in the fluid and creates a holding force against droplet spreading. Droplet fluid force of inertia remains high at high Weber numbers, and it gives rise to increase spread factor. This is observed for pure water droplet, *i.e.* increasing Weber number causes the increase spread factor. Hence, magnetic force reduces the force of inertia even for small concentration of ferro particles (0.005%), which lowers the spreading factor at large numbers. The contact time reduces gradually for ferro particle droplets as compared to water droplet as Weber number increases. Due to increased fluid inertia at high Weber numbers, droplet spreading and retraction durations reduce. However, magnetic field creates a magnetic force lowering both spreading and contraction of the droplet and rebounding initiation starts earlier than the pure water droplet. This becomes more visible for large ferro particle concentration (0.05% wt). Fig. 6 shows vertical displacement (rebound height) of droplet after impact for two ferro particles concentrations. Although the droplet fluid mass reduces over the mesh surface after initial impact due to impalement, droplet undergoes rebounding on the surface, which is more pronounced for pure water and low ferro particle concentration. This is associated with the separation of ferro particles with some droplet fluid, which are pull-off from the droplet by the magnetic force. The separated fluid with high concentration of ferro particles penetrates through the mesh screens due to the pull force of the magnet in the gravitational direction. Therefore, droplet fluid remaining above the mesh surface has less ferro particles and influence of magnetic force on the droplet rebounding almost ceases. This can clearly be observed from the behavior droplet with low concentration ferro particles, which have higher displacement height than that of droplet with high concentration particles. Moreover, the suppression of rebound height of droplet by magnetic field *t* is evident, since pure water has the highest displacement height as compared those of droplets with ferro particles mixed.

**Fig. 7:**
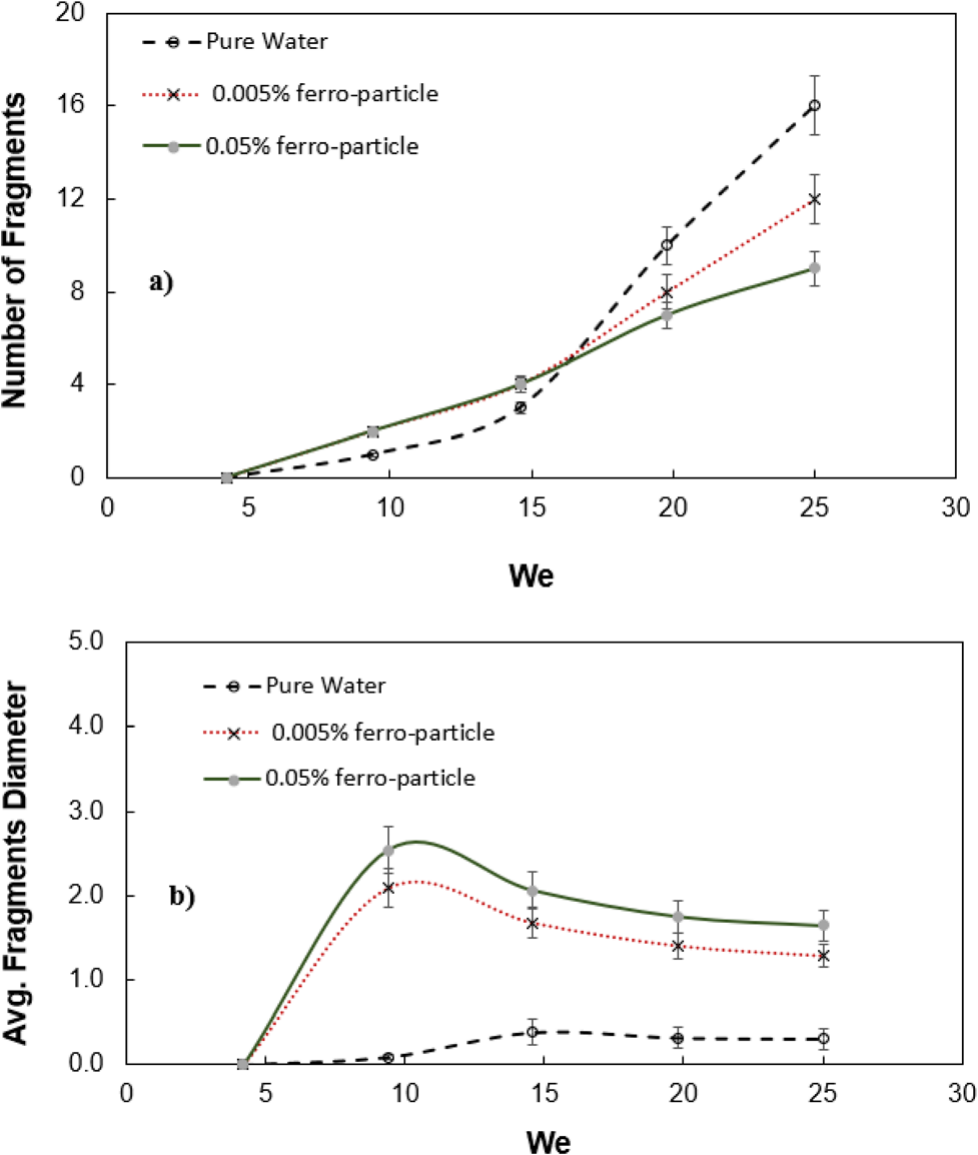
Fragmented droplet characteristics with Weber number: (a) number of fragmented droplets, and (b) average size of fragmented droplets.

Fig. 7a shows the number of droplets fragmented from impacting droplets after impalement under the magnetic force. The pure water droplet is also included for comparison. Droplet fragmentation from the mesh and number of newborn droplet formation increase at higher Weber numbers. However, the droplet fragmentation for pure water droplet increases significantly as compared to ferro particle mixed droplet for Weber number larger than 15. The magnetic force slows down the fragmentation of the droplet for large Weber numbers. Impact early stage, some droplet volume undergoes impalement in impact region of the mesh and the radial droplet momentum enables droplet spread on the mesh surface. During the spreading period droplet impalement remains low and droplet fragmentation replaces the fluid spreading over the mesh surface. As indicated earlier, the capillary and the magnetic forces create a blockage effect preventing droplet fluid impalement while lowering the number of droplet fragments. The shapes of the fragmented droplets change towards the elongated droplets (Fig. 2) under the magnetic influence. Fig. 7b shows the average diameter of the fragmented droplets. Average diameter of the fragmented water droplets is also given for comparison. Average diameter of the fragmented water droplets after the impact increases up to Weber number about 12 and remains almost same with increasing Weber number. At low Weber numbers the amount of liquid penetration into mesh is less, which gives rise to small amount of fluid impalement and causing a small impalement fluid droplet diameter. However, at large Weber numbers (We > 12), the amount of fluid impalement increases, yet the penetrated liquid droplet diameter remains almost same, *i.e.* fluid undergoes break off large number of droplets with almost same size due to large number of fragments (Fig. 7a).

## 4 Conclusion

A droplet composing of water ferro-particle mixture and impacting on hydrophobic mesh is considered and droplet dynamics after impact is examined. Two-mesh sizes and two ferro-particle concentrations are incorporated, and magnetic influence is introduced in the experiments. The meniscus height of implement fluid and restitution coefficient of droplet on mesh surface are formulated and findings are compared with the experimental data. It is found that the restitution coefficient predicted agrees with the experimental data. Some volume of impacting droplet fluid undergoes penetration into the mesh and breaks off to newborn droplets. The volume fraction of impalement liquid increases with increasing Weber number, which signifies with applied magnetic field in the direction of gravity. Increasing concentration of ferro particles in the water mixture reduces droplet contact duration on the mesh surface upon impact while slightly enhances the spread factor number of droplet fragments, which is more pronounced at high Weber numbers. The restitution coefficient reduces notably with increasing Weber number, *i.e.* it is about 0.58 for We = 5 and increases to almost 0.23 for We = 25 for the mesh aspect ratio of 64.5%. The implementation of fluid fragments has given rise to new droplets. The number and diameter of the newborn droplets becomes large with increasing Weber number and concentrations, which is more pronounced for pure water droplets. In this case, number of fragmentations for pure water is 16 for We = 25 and it is only 8 for 0.05% ferroparticle concentration droplet at the same Weber number. The influence of mesh size on droplet dynamic properties in terms of contact duration, percentage of volume penetration, and number of droplet fragments is more significant than the ferro particle concentration in the water mixture. Moreover, these properties attain large values for bigger screen aspect ratio and the influence of aspect ratio becomes notable as the Weber number increases, particularly for percentage of volume penetration. In this case, volume penetration is 11% for We = 5 while it increases to 95% for We = 25. In summary, our results demonstrate that magnetic forces, increasing Weber number, and larger mesh sizes significantly enhance liquid impalement and energy dissipation, thereby reducing the restitution coefficient and altering the overall droplet behavior. This study gives insight into water mixed ferro particle droplet impact characteristics under magnetic influence and provides useful information on the contribution of the magnetic field to the impacting droplet behavior on hydrophobic mesh surfaces.

## Author contributions

B. S. Yilbas did the research work with the collaboration of other co-authors and wrote the manuscript. Ghassan Hassan did experimental and analytical works and contributed to the writing of the manuscript. A. Al-Sharafi did some part of the experimental work and contributed to the writing of the manuscript. Abba Abdulhamid Abubakar some part of the analysis and contributed to the writing of the manuscript. H. Al-Qahtani did some part of the analysis and analytical work and contributed to the writing of the manuscript.

## Conflicts of interest

The author declares that there is no conflict of interest.

## Data Availability

The datasets used and/or analyzed during the current study are available from the corresponding author on reasonable request.
